# Richard Green, M.D., J.D. (1936–2019)

**DOI:** 10.1007/s10508-019-01474-3

**Published:** 2019-06-04

**Authors:** Joe Herbert

**Affiliations:** John van Geest Centre for Brain Repair, Department of Clinical Neurosciences, University of Cambridge, Cambridge, CB2 0PY England UK

It was Winston Churchill who once wrote: We make a living by what we get; we make a life by what we give.

Richard Green gave many things to those who knew him and to the world he lived in. He had the marvelous combination of a high intellect, scientific entrepreneurship, enormous moral courage, and deep concern about people whom he thought were disadvantaged because of what they were, rather than who they were. He had some personal experience of this. Anti-Jewish sentiment was more institutionalized when Richard was young than it is now: he was one of the six of the annual Jewish quota then allowed into Johns Hopkins Medical School. He could easily have been distinguished in one of the more traditional, and safer, paths of medicine. Specializing in sexuality is still thought of as something of a psychiatric sideshow and, by some, not entirely reputable.

**Fig. 1:**
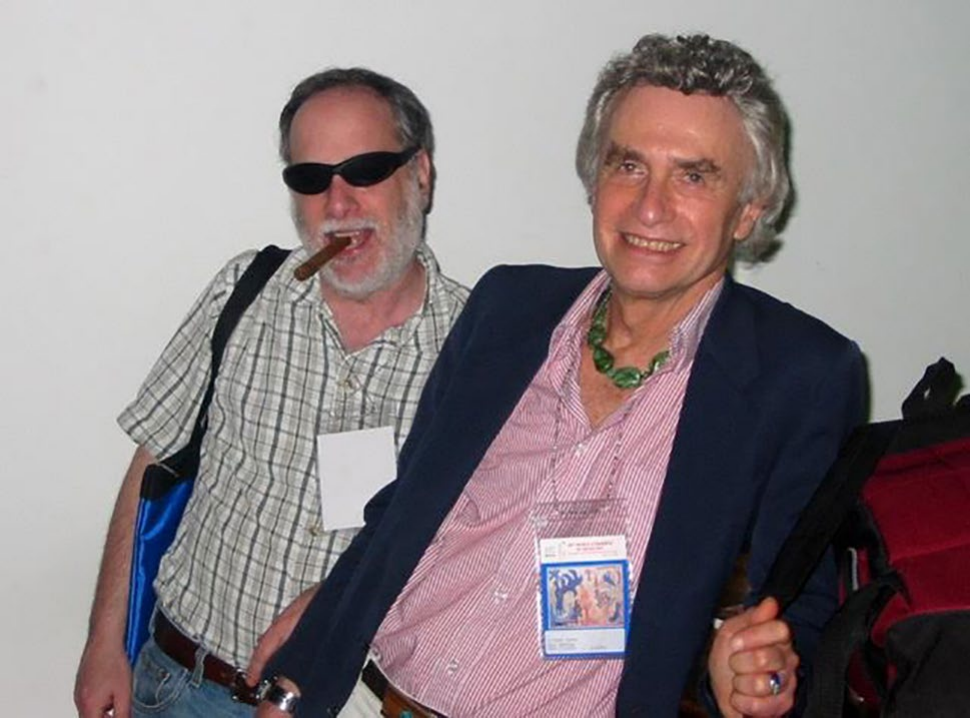
Richard Green, M.D., J.D. (Editor of *Archives of Sexual Behavior*, 1971–2001), with Ken Zucker, Ph.D. (Editor of *Archives of Sexual Behavior* (2002-present), at the 2003 meeting of the World Congress of Sexology in Havana, Cuba. Photograph courtesy of Alain J. Giami, Ph.D.

Extraordinary, when one considers the impact and significance that sex has in all our lives and the behavior of our societies. But working in this area requires multiple skills: clinical expertise, extensive biological knowledge and the willingness and an ability to cross disciplinary borders, familiarity with aspects of the law, a nose for political debate and the skills to take part; but most of all, courage. Courage to challenge the contemporary views on sexuality, how it is viewed, and how society treats it. Courage to challenge contemporary clinical methods of dealing with aberrant or distressing sexuality. Courage to defy rejection from grant-giving bodies or academic institutions, but to persevere until success comes your way. Courage to become a target for political zealots of various colors. Courage to interrupt a high profile career, return to a high-ranking university in middle age as a student; and outperform a bunch of over-ambitious high-flying 20-somethings. Richard had courage (Fig. 1).

Richard entered a society that considered homosexuality a sin and a crime, same sex marriages as unimaginable, transgender people as freaks, and lesbian or gay parents as totally unsuitable. From where we now stand, such attitudes seem unimaginably unacceptable (though there are still parts of the world who maintain them). A few years ago, a member of the UK parliament “came out” as gay. Fifty years ago, that person’s career would have ended. But when this happened, it was considered minor news, and most people shrugged and said “so what?” An openly gay man is running for President of the U.S. in 2020. Of course, no one person is responsible for the dramatic change in (some) societies’ views on these matters, but Richard was in the forefront. His reputation as a clinician and a scientist ensured that people listened to him, even when they disagreed. In hindsight, being a pioneer seems like fun, exhilarating, and rewarding: at the time, it required bravery, clear vision and determination, and a very thick skin. The improving tolerance we enjoy today, still imperfect, we owe to people like Richard.

As John Maynard Keynes (almost) said: A good scientist changes her/his/their mind when the facts change. One of Richard’s major scientific achievements was a long-term study of young boys with markedly feminine gender-variant behavior, including the wish to be a girl (Green, 1974, 1987). This was supposed to be the origin of transgender. But these lads didn’t change gender—the majority of them became gay. Like all good scientists, Richard—instead of an obstinate retention of the original idea—revised his interpretation. Green’s (1987) book is a model of elegant writing and interesting data. This was the beginning of a long series of influential studies on therapeutic approaches to various forms of gender identity or sexual identity conflict, but also on the psychological and psychiatric consequences of being gay, a subject that was hardly studied 50 years ago. This led Richard into social and legal aspects of being either gay or transgender. Most of those in his position would have tried to pick up some smatterings of law or collaborated with lawyers. How typical of Richard’s energy, adventurousness, and self-belief that he decided to attend Yale University as a law student. Naturally, he excelled. He returned to his comprehensive approach to his subject with another arrow in his packed quiver (Green, 1992).

And comprehensive it was. Richard was not a bystander. He decided that the scientific literature needed a journal devoted to sexuality in all its forms: so he founded one, and edited it for 30 years. The *Archives of Sexual Behavior* is now one of the leading journals in its field. He decided that sexuality was such a diverse field that it needed a forum where scientists and clinicians who were unlikely to meet might exchange views: so he founded one. The International Academy of Sex Research has now has an international presence and has met every year since 1975; Richard was its first president. Since 2016, the annual Richard Green lecture treasures his life and achievements and the *Journal* has launched an annual award in Richard’s name for the best publication in the calendar year (Zucker, 2019).

These are lasting tributes to his massive and unique contribution to his field. It doesn’t stop there. He initiated major and important debates about the optimum ways of helping those with gender dysphoria or improving social attitudes to gay or transgender persons, amid increasingly strident political clamor. He was a skilled and persuasive communicator on radio and television, and in the popular press. Some of these skills he would have learned as a (very good-looking) summer theater actor in his youth (he never lost his interest in showbiz, and he had some glamorous friends). In all these ways, and more, he promoted his subject, graced his discipline, and helped make a resounding and lasting improvement in attitudes to sexuality. He attracted friends and devoted support by Melissa Hines and, more latterly, by Claire Lovejoy. He was fun, he was great company (though occasionally infuriating), he was a fount of knowledge and stories about sexuality in all its forms and variety and, lastly, I am deeply proud to say: for about 50 years he was my friend.
